# Predicting intrahepatic recurrence of colorectal cancer liver metastases after curative hepatectomy using a machine learning model with data integration of ultrasound radiomics and clinicopathological parameters

**DOI:** 10.1186/s13244-026-02227-2

**Published:** 2026-03-16

**Authors:** Ting Hu, Zhong Liu, Pengpeng Kuang, Yunyun Li, Weixuan Kong, Wei Zheng, Jianjun Li, Guangjian Liu, Han Zhang, Xin Chen, Ruhai Zou

**Affiliations:** 1 Department of Endoscopy, State Key Laboratory of Oncology in South China, Guangdong Provincial Clinical Research Center for Cancer, Sun Yat-Sen University Cancer Center, Guangzhou, P. R. China; 2 National-Regional Key Technology Engineering Laboratory for Medical Ultrasound, Guangdong Key Laboratory for Biomedical Measurements and Ultrasound Imaging, School of Biomedical Engineering, Shenzhen University, Shenzhen, P. R. China; 3 Guangdong Lung Cancer Institute, Guangdong Provincial Key Laboratory of Translational Medicine in Lung Cancer, Guangdong Provincial People’s Hospital, Guangdong Academy of Medical Sciences, Southern Medical University, Guangzhou, China; 4 Department of Medical Ultrasonics, The Sixth Affiliated Hospital, Sun Yat-sen University, Guangzhou, P. R. China; 5 Department of Medical Ultrasonics, Second Affiliated Hospital of Kunming Medical University, Kunming, P. R. China; 6 Department of Ultrasound, State Key Laboratory of Oncology in South China, Guangdong Provincial Clinical Research Center for Cancer, Sun Yat-Sen University Cancer Center, Guangzhou, P. R. China; 7 Institute of Acoustics, Chinese Academy of Science, Beijing, P. R. China

**Keywords:** Colorectal cancer liver metastases, Ultrasound radiomics, Intrahepatic recurrence, Machine learning

## Abstract

**Objectives:**

To develop and validate a machine learning model integrating ultrasound radiomics and clinicopathological parameters to predict intrahepatic recurrence in colorectal cancer liver metastases (CRLM) patients after curative hepatectomy.

**Materials and methods:**

This retrospective study enrolled 278 eligible CRLM patients (age, 55 ± 12 years; male, 188) from two centers, including a main cohort (*n* = 224, July 2010–February 2021) and an external cohort (*n* = 54, February 2015–October 2020). Patients were stratified by recurrence status during a 2-year follow-up. Preoperative ultrasound images and clinicopathological parameters were collected. Radiomics features were extracted from liver metastases, peri-tumor areas, and disease-free liver parenchyma. Using least absolute shrinkage and selection operator (LASSO) analysis and support vector machine algorithms, three predictive models were developed: clinical, radiomics, and clinical-radiomics combined (cRadiomics) models. Model performance was assessed using five-fold cross-validation (main cohort) and external validation (external cohort), with metrics including receiver operating characteristic (ROC) curve, the area under the ROC curve (AUC), accuracy, sensitivity, and specificity.

**Results:**

Six clinical parameters (pathological lymph node positivity, synchronous liver metastases, bilobar liver metastases, preoperative chemotherapy, use of targeted drugs, and preoperative CA19-9 > 200 U/mL) and seven radiomics features were identified as strong predictors. The cRadiomics model achieved AUC values of 0.811 (95% CI: 0.755–0.861) and 0.784 (95% CI: 0.644–0.880) during testing on the main cohort and external cohort data, respectively, significantly outperforming both radiomics (AUC 0.744 and 0.724; *p* < 0.01) and clinical models (AUC 0.706 and 0.696; *p* < 0.05).

**Conclusions:**

The cRadiomics model, integrating ultrasound radiomics and clinicopathological parameters, improved the prediction of intrahepatic recurrence within two years for colorectal liver metastases after curative hepatectomy.

**Critical relevance statement:**

The cRadiomics model, with enhanced accuracy in predicting intrahepatic recurrence of colorectal liver metastases after curative hepatectomy, holds great potential to improve clinical decision-making and enable personalized management and risk-adapted follow-up for colorectal cancer liver metastases (CRLM) patients.

**Key Points:**

- The cRadiomics model can predict intrahepatic recurrence of colorectal cancer liver metastases (CRLM) after curative hepatectomy.
- Machine learning with data integration of ultrasound radiomics and clinicopathological parameters enables better prediction of intrahepatic recurrence for CRLM after curative hepatectomy.
- The developed model holds great potential to improve clinical decision-making and personalized management for CRLM patients.

**Graphical Abstract:**

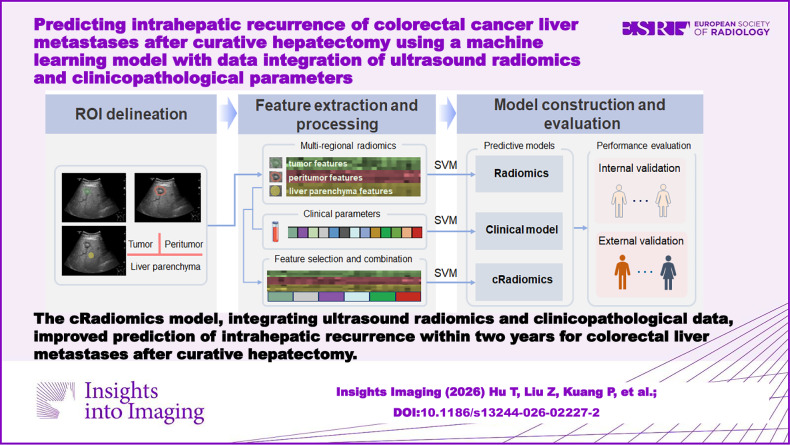

## Introduction

Colorectal cancer (CRC) is the third most commonly diagnosed cancer worldwide and the second leading cause of cancer-related mortality [1]. The liver is the preferred site of metastasis for CRC due to its anatomical proximity and connection to the portal systemic circulation. Nearly 50% of patients develop liver metastases during the course of the disease [2, 3]. Therapeutic options for CRC liver metastases (CRLM) include hepatectomy, systemic chemotherapy, radiotherapy, hepatic artery embolization, and thermal ablation techniques, such as microwave coagulation therapy and radiofrequency ablation [4]. Hepatectomy remains the standard treatment for CRC liver metastases (CRLM), providing long-term survival and potential cure in 20%–50% of patients who undergo complete resection [5]. However, previous studies have shown that only 10%–20% of patients with CRLM are initially eligible for curative resection [6, 7]. For resectable liver metastases, patients should receive perioperative chemotherapy, which comprises three months of FOLFOX4 (5-fluorouracil, folinic acid, and oxaliplatin) before surgery, followed by another three months postoperatively. For unresectable liver metastases, neoadjuvant chemotherapy is administered to convert initially unresectable CRLM into resectable cases [8, 9]. However, prolonged chemotherapy can result in chemotherapy-induced liver injury, such as hepatic steatosis, which can impair the sensitivity of preoperative imaging modalities in detecting lesions [10, 11]. Despite therapeutic advancements having reduced the risk of recurrence after surgery, approximately 50%–70% of patients still experience recurrence, with the majority occurring within two years post-resection. One-third of these patients have a recurrence confined to the liver [12–14]. This high recurrence rate is associated with a reduced 5-year survival rate of only 37%–58% [15]. Thus, timely diagnosis and reliable recurrence prediction are critical for precision medicine and personalized follow-up, thereby ultimately improving outcomes and quality of life.

Radiomics extracts a wide range of quantitative features from medical images to detect subtle microstructural changes within tumors that are hardly perceptible to the naked eye, providing insights correlating with tumor biology, treatment response, potential recurrence risk, and prognosis [16, 17]. Based on preoperative whole-liver enhanced computed tomography (CT) images, a radiomics model showed promising performance [18] for predicting metachronous metastases within two years following rectal cancer surgery. Simpson et al demonstrated that radiomics characteristics based on CT may identify patients at risk of recurrence after hepatectomy, including early CRLM recurrence within 6 months [19, 20].

Recent studies report that ultrasound-based radiomics, as a promising branch of radiomics, has been applied to assess focal liver lesions, such as hepatocellular carcinoma [21], liver metastases [22], and diffuse hepatic diseases [23]. This study aims to develop and validate a machine learning model to predict intrahepatic recurrence in patients with colorectal liver metastases after curative hepatectomy. The model integrates ultrasound radiomics features from tumors, peritumoral regions, and grossly disease-free liver parenchyma with clinicopathological parameters. The findings of this study may contribute to improving clinical decision-making and enabling personalized management and risk-adapted follow-up for CRLM patients.

## Materials and methods

### Study population

**Fig. 1:**
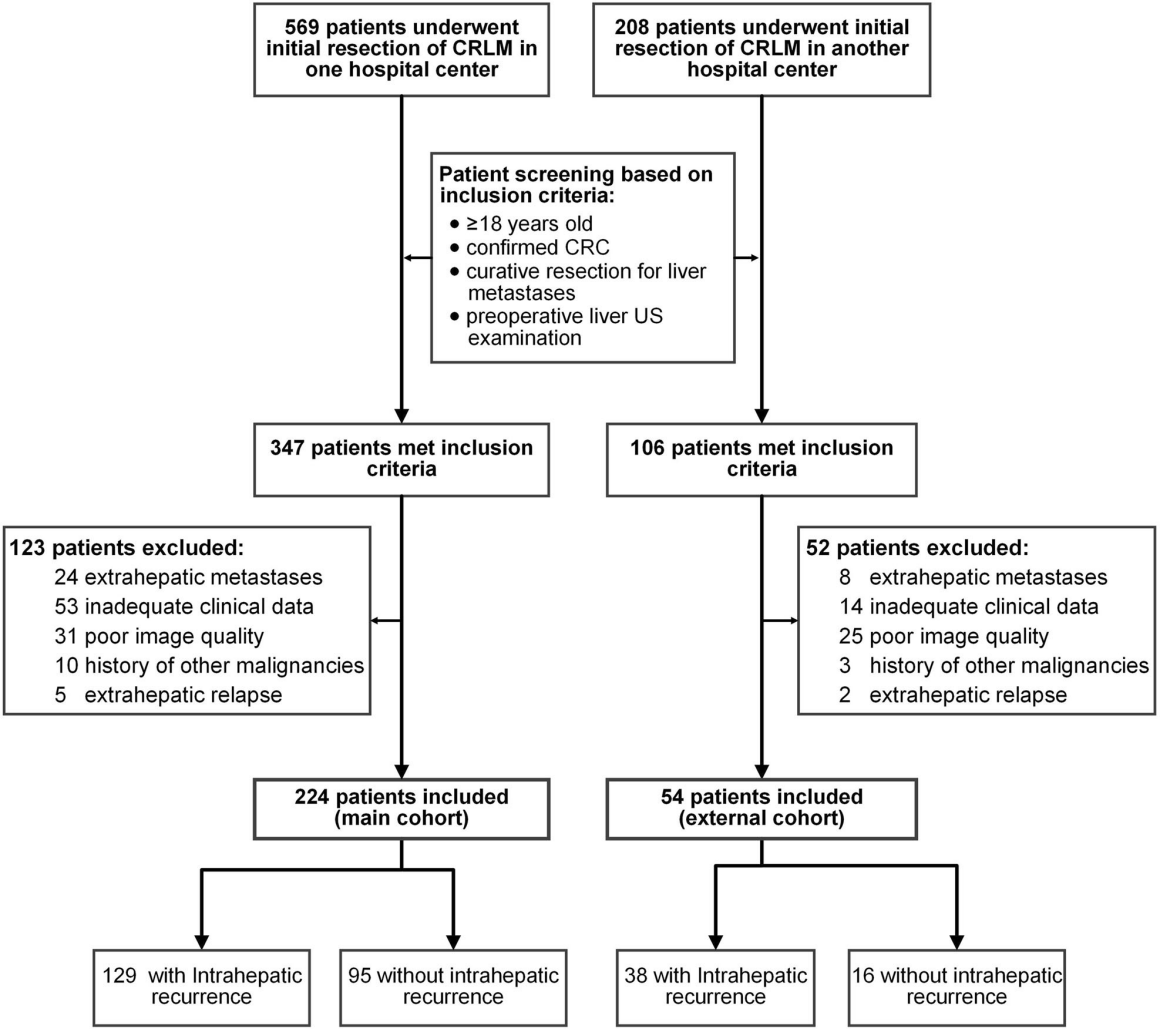
Workflow for patient screening

This study was conducted in accordance with the Helsinki Declaration and was approved by the Medical Ethics Committee of Sun Yat-Sen University Cancer Center (No. B2023-605-01). Informed consent for this retrospective study was waived due to its observational design. However, each participant provided written informed consent for the Collection and Application of Clinical Samples and Medical Data, certified and approved by the ethics committee of Sun Yat-Sen University Cancer Center upon hospital admission. Consecutive patients who underwent initial resection of CRLM between July 2010 and February 2021 were retrospectively recruited from the clinical database of Sun Yat-Sen University Cancer Center (SYSUCC, Guangzhou, China). Among these patients, eligible individuals were selected to form the main cohort for model training and internal evaluation. The inclusion criteria were: (1) age ≥ 18 years old, (2) pathologically confirmed colorectal adenocarcinoma, (3) curative resection for the liver metastases, and (4) a preoperative liver ultrasound examination. The exclusion criteria were: (1) extrahepatic metastases to the lung, bone, brain, peritoneum, and distant lymph nodes, (2) inadequate clinical information, (3) poor quality of ultrasound images, (4) extrahepatic relapse, and (5) history of other malignancies. From February 2015 to October 2020, eligible patients from the Sixth Affiliated Hospital of Sun Yat-Sen University (SYSUASH, Guangzhou, China) were identified based on the same criteria, constituting an independent cohort for external validation of the model derived from the main cohort. A flowchart illustrating the patient screening process is presented in Fig. 1.

### Clinical data collection

Clinical data for patients with CRLM, including patient-related factors, primary tumor factors, liver metastasis factors, and treatment-related factors, were retrospectively collected from medical records. Synchronous liver metastases were defined as those detected at or before the diagnosis of the primary lesion, while metachronous metastases were identified as those detected after CRC surgery [24]. Some CRLM patients underwent not only hepatectomy but also intraoperative open ablation. “Complete ablation” was defined as the absence of enhancement in the entire ablated area on follow-up contrast-enhanced CT or magnetic resonance imaging (MRI) at 1 month post-ablation [25]. Preoperative levels of carcinoembryonic antigen (CEA) and carbohydrate antigen 19-9 (CA19-9) were obtained from laboratory tests. Intrahepatic recurrence, defined as the detection of new intrahepatic lesions or metastases with imaging features typical of colorectal liver metastasis on abdominal contrast-enhanced CT or contrast-enhanced MRI, or atypical findings confirmed by histopathological examination, within two years of curative resection.

### Ultrasound image acquisition and preprocessing

Preoperative liver grayscale ultrasound images in DICOM format, acquired using Philips IU22 (Philips Medical Systems), GE Logiq E9 (GE Healthcare), and similar diagnostic instruments, were also collected from the medical recording system. Under the supervision of a senior radiologist with 10 years of ultrasound experience, a junior radiologist with 3 years’ experience delineated the tumor, peritumoral, and liver parenchyma region of interest (ROI) for all patients. For each lesion, an ROI was manually delineated around the tumor boundary on the grayscale ultrasound image of the largest cross-section. A peritumoral ROI was automatically expanded by 1 cm from the lesion boundary, while a parenchymal ROI with a specified diameter (2 cm) was manually plotted in areas of the liver parenchyma, ensuring that the selected area was free of visible disease or anatomical structures. Cases for which a 1 cm peritumoral expansion was infeasible, because of the proximity to the liver capsule or other anatomical boundaries, were excluded. All ROIs were reviewed by the senior radiologist, and adjustments were made as necessary to ensure accurate delineation. Subsequently, all images underwent standardized preprocessing, including intensity discretization using a fixed bin width. Radiomics features were extracted using the PyRadiomics extension in 3D Slicer (version 5.0.3) and standardized using z-score to ensure comparability across patients.

### Model development

**Fig. 2:**
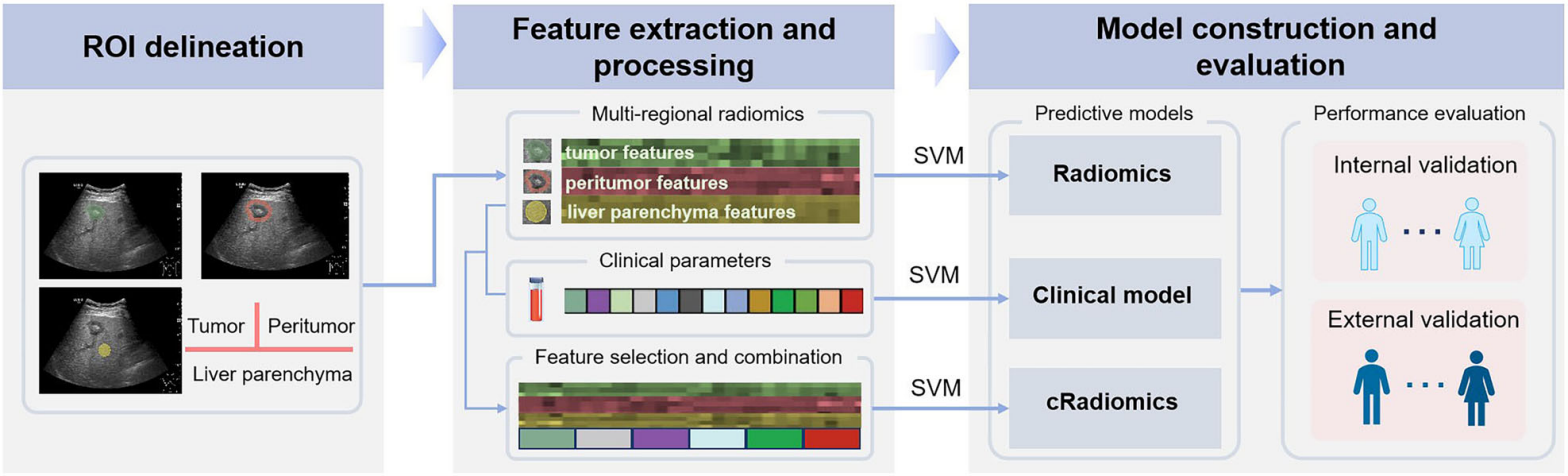
Workflow for model development and evaluation

Prognostic models were established using data from the main cohort. During model development, radiomics features and clinicopathological parameters, were screened using least absolute shrinkage and selection operator (LASSO) in order to identify strong predictors of intrahepatic recurrence. Spearman analysis was performed to assess the correlations between each pair of feature variables screened out by LASSO, and those between the variables and the outcome of recurrence, with the sole purpose of evaluating both the utility of the LASSO-selected features and their redundancy. Thereafter, the machine learning algorithm of support vector machine was used to build three prognostic models: a clinical model based on clinicopathological parameters, a radiomics model based on ultrasound radiomics features, and a combined model (cRadiomics) integrating both. Figure 2 presents an overview of the model development procedure.

### Model evaluation

Both internal and external validations were performed to evaluate the models’ classification performances. For internal validation, twenty-five-fold cross-validation (FFCV) was performed on the main cohort (Multiple runs of FFCV enable robust comparisons, as each run involves random data splitting, and repeating the process accounts for such randomness to enhance result reliability.). After completing twenty rounds of FFCV, the predicted probabilities for samples involved in internal training or testing of FFCV were aggregated together to generate a composite receiver operating characteristic (ROC) curve [26], from which a set of quantitative metrics, including accuracy, sensitivity, specificity, and the area under the ROC curve (AUC), were calculated for performance evaluation. For external validation, all models were retrained on the entire main cohort data; the resulting models were then evaluated on the external cohort using metrics identical to those of internal validation (Since multiple models were trained on the main cohort across repeated FFCV runs during internal validation, these internal validation-derived models were excluded from the external test to alleviate storage and computational burdens in real-world deployment scenarios. Instead, retraining on the entire main cohort produced a single model for external validation—a process that mirrors real-world practices, ensuring efficient model development and deployment. See supplementary material for more details on the internal/external validations. Apart from assessing the models’ classification performance, we also evaluated their calibrations by means of calibration plots, calibration slope/intercept, and Brier score [27].

### Statistical analysis

Continuous clinical variables were presented as mean ± standard deviation and compared using the Mann–Whitney *U*-test, while categorical variables were shown as frequencies (proportion, %) and analyzed using the Chi-square test, except preoperative CEA (< 200 or > 200 ng/mL), which was assessed using Fisher’s exact test. Spearman analysis was conducted to assess the correlations between each pair of feature variables screened out by LASSO, and those between the variables and the outcome of recurrence. The 95% confidence intervals (CIs) for the accuracy, sensitivity, specificity, and AUC were computed using the Clopper–Pearson method [28]. The DeLong test was used to measure the significance of differences between AUC values. A two-tailed p-value less than 0.05 was considered statistically significant. Data processing and model development were conducted using scikit-learn (version 0.23.2) in Python (version 3.8.1).

## Results

### Patient characteristics

A total of 224 patients (age, 54 ± 11 years; male 154) from SYSUCC were included. The main cohort of patients consisted of 154 (68.8%) males and 70 (31.2%) females, all of whom underwent curative liver resection with or without additional intraoperative open ablation. By the end of follow-up, 129 (57.6%) patients experienced intrahepatic relapse, while 95 (42.4%) showed no evidence of relapse. Patients with intrahepatic recurrence were more likely to present with synchronous liver metastases (*n* = 109, 84.5% vs *n* = 63, 66.3%; *p* = 0.001), bilobar liver metastases (*n* = 68, 52.7% vs *n* = 21, 22.1%; *p* < 0.001), and a tumor number ≥ 3 (*n* = 79, 61.2% vs *n* = 37, 38.9%; *p* = 0.001). These patients also tend to receive adjuvant chemotherapy (*n* = 122, 94.6% vs *n* = 74, 77.9%; *p* < 0.001), targeted therapies (*n* = 57, 44.2% vs *n* = 25, 26.3%; *p* = 0.006), and intraoperative ablation (*n* = 48, 37.2% vs *n* = 19, 20%; *p* = 0.005). Furthermore, pathological lymph node positivity (N1–N2) (*n* = 75, 58.2% vs *n* = 37, 38.9%; *p* = 0.005) and preoperative CA19-9 > 200 U/mL (*n* = 13, 10.1% vs *n* = 2, 2.1%; *p* = 0.018) were common in patients with intrahepatic recurrence. No significant differences were observed between the two groups in terms of age, gender, primary tumor location, maximum diameter of CRLMs, and other clinical variables (all *p* > 0.05). Due to a relatively high proportion of missing values (23.0%) of microsatellite instability (MSI) across both cohorts, it was not included in groupwise statistical comparisons or multivariable modeling.

By screening patients from SYSUASH, a total of 54 patients (age, 58 ± 13 years; male 34) were included as the external cohort, among which 51 patients were diagnosed as synchronous CRLM, and 44 patients underwent simultaneous resection. Most patients (74.1%) had a tumor number <3. Thirty-eight patients experienced intrahepatic relapse within two years after liver resection. Patient baseline characteristics are summarized in Table 1.

**Table 1:** Baseline characteristics for patients in the main and external cohorts

Variable	Main cohort				External cohort			
	Total	Intrahepatic recurrence (*n* = 129)	Without intrahepatic recurrence (*n* = 95)	*p*	Total	Intrahepatic recurrence (*n* = 38)	Without intrahepatic recurrence (*n* = 16)	*p*
Age, years	224	53 ± 12 (25–73)	55 ± 11 (29–83)	0.203	54	58 ± 13 (27–81)	59 ± 11 (38–81)	0.631
Age				0.583				0.808
≤ 60	146	90 (69.8)	63 (66.3)		29	20 (52.6)	9 (56.3)	
> 60	78	39 (30.2)	32 (33.7)		25	18 (47.4)	7 (43.7)	
Sex				0.623				0.964
Male	154	87 (67.4)	67 (70.5)		34	24 (63.2)	10 (62.5)	
Female	70	42 (32.6)	28 (29.5)		20	14 (36.8)	6 (37.5)	
Primary tumor location				0.239				0.843
Right colon	42	27 (20.9)	15 (15.8)		22	18 (47.4)	4 (25)	
Left colon	94	48 (37.2)	46 (48.4)		9	4 (10.5)	5 (31.3)	
Rectum	88	54 (41.9)	34 (35.8)		23	16 (42.1)	7 (43.7)	
Timing of liver metastasis				**0.001**				0.547
Synchronous	172	109 (84.5)	63 (66.3)		51	35 (92.1)	16 (100)	
Metachronous	52	20 (15.5)	32 (33.7)		3	3 (7.9)	0	
Number of CRLMs				**0.001**				0.515
< 3	108	50 (38.8)	58 (61.1)		40	27 (71.1)	13 (81.3)	
≥ 3	116	79 (61.2)	37 (38.9)		14	11 (28.9)	3 (18.7)	
Maximum diameter of CRLMs				0.859				0.306
< 3 cm	112	66 (51.2)	46 (48.4)		37	24 (63.2)	13 (81.3)	
3–5 cm	71	39 (30.2)	32 (33.7)		13	10 (26.3)	3 (18.7)	
> 5 cm	41	24 (18.6)	17 (17.9)		4	4 (10.5)	0	
Location of CRLMs				< **0.001**				0.08
Unilobar	135	61 (47.3)	74 (77.9)		36	22 (57.9)	14 (87.5)	
Bilobar	89	68 (52.7)	21(22.1)		18	16 (42.1)	2 (12.5)	
Simultaneous resection				0.247				0.249
No	146	80 (62)	66 (69.5)		10	9 (23.7)	1 (6.3)	
Yes	78	49 (38)	29 (30.5)		44	29 (76.3)	15 (93.7)	
Preoperative chemotherapy				< **0.001**				0.947
No	28	7 (5.4)	21 (22.1)		24	17 (44.7)	7 (43.7)	
Yes	196	122 (94.6)	74 (77.9)		30	21 (55.3)	9 (56.3)	
Use of targeted drugs				**0.006**				0.474
No	142	72 (55.8)	70 (73.7)		43	29 (76.3)	14 (87.5)	
Yes	82	57 (44.2)	25 (26.3)		11	9 (23.7)	2 (12.5)	
Intraoperative ablation				**0.005**				1
No	157	81 (62.8)	76 (80)		53	37 (97.4)	16 (100%)	
Yes	67	48 (37.2)	19 (20)		1	1 (2.6)	0	
Pathological T stage				0.38				0.342
1-2	39	20 (41.9)	19 (20)		37	28 (73.7)	9 (56.3)	
3-4	185	109 (58.1)	76 (80)		17	10 (26.3)	7 (43.7)	
Pathological N stage				**0.005**				0.3
0	112	54 (41.8)	58 (61.1)		17	13 (34.2)	4 (25)	
1–2	112	75 (58.2)	37 (38.9)		37	25 (65.8)	12 (75)	
High MSI				NA				NA
No	160	100 (77.5)	60 (63.2)		49	36 (94.7)	13 (81.3)	
Yes	4	2 (1.6)	2 (2.1)		1	0	1 (6.3)	
Unknown	60	27 (20.9)	33 (34.7)		4	2 (5.3)	2 (12.4)	
Preoperative CEA, ng/mL				0.507				
≤ 5	69	42 (32.6)	27 (28.4)		32	21 (55.3)	11 (68.7)	0.357
> 5	155	87 (67.4)	68 (71.6)		22	17 (44.7)	5 (31.3)	
Preoperative CA19-9, U/mL				0.905				0.751
≤ 35	173	100 (77.5)	73 (76.8)		38	26 (68.4)	12 (75)	
> 35	51	29 (22.5)	22 (23.2)		16	12 (31.6)	4 (25)	
Preoperative CEA, ng/mL				0.243*				NA
≤ 200	217	123 (95.3)	94 (98.9)		54	38 (100)	16 (100)	
> 200	7	6 (4.7)	1 (1.1)		0	0	0	
Preoperative CA19-9, U/mL				**0.018**				0.306
≤ 200	209	116 (89.9)	93 (97.9)		49	33 (86.8)	16 (100)	
> 200	15	13 (10.1)	2 (2.1)		5	5 (13.2)	0	

Continuous and categorical variables were compared using the Mann–Whitney *U*-test and chi-square test, respectively. The asterisk denotes that Fisher’s exact test was utilized. “Unknown” MSI status indicates missing or unrecorded data. Bold values indicate statistically significant differences (*p* < 0.05)

*CRLM* colorectal cancer liver metastases, *CAE* carcinoembryonic antigen, *CA19-9* carbohydrate antigen 19-9, *NA* not applicable, *MSI* microsatellite instability

### Selected features

**Fig. 3:**
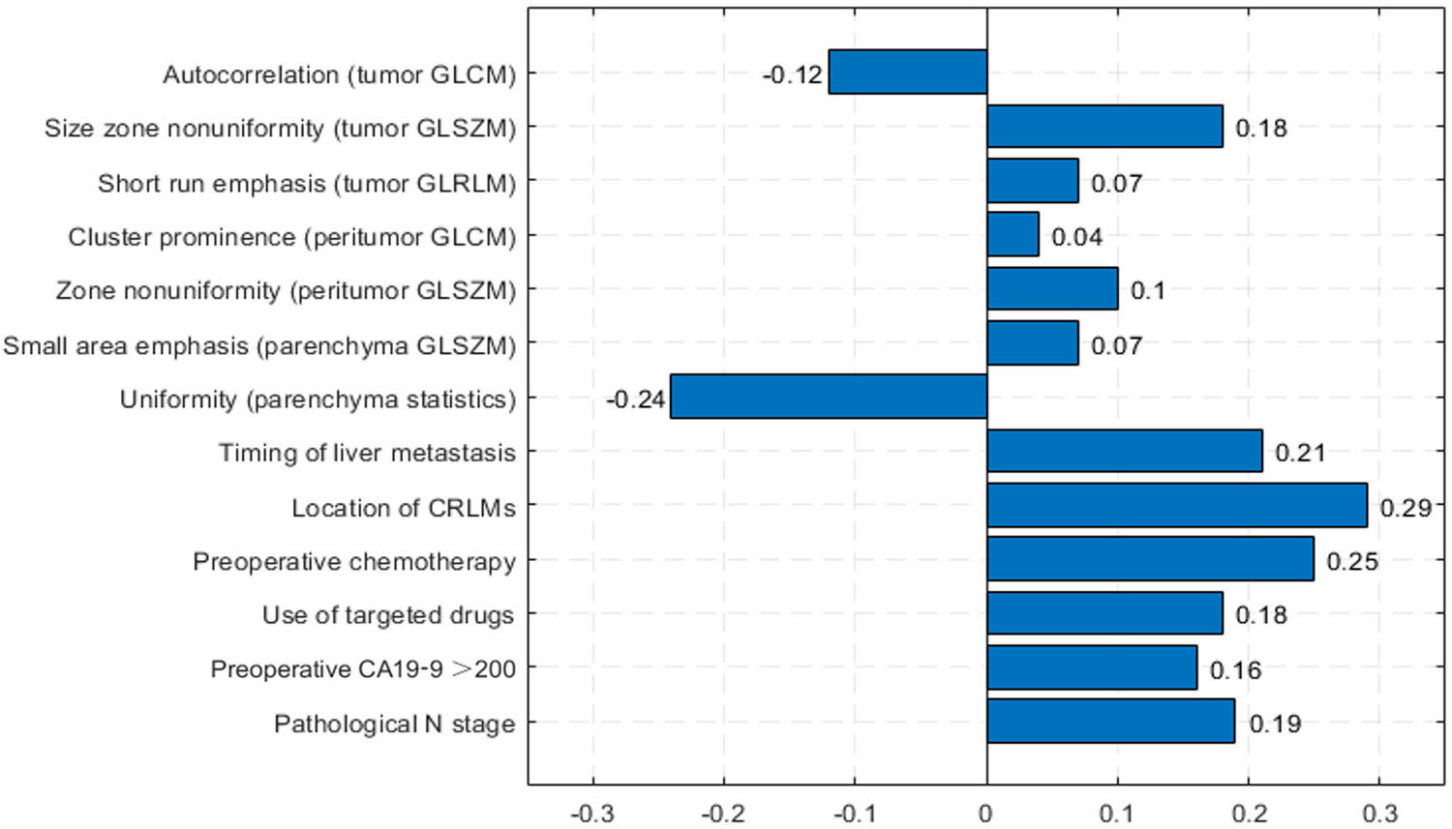
Bar plots of the coefficients for correlations between intrahepatic recurrence and selected features according to Spearman correlation analysis. GLCM, gray-level co-occurrence matrix; GLSZM, gray-level size zone matrix; GLRLM, gray-level run length matrix; CRLM, colorectal cancer liver metastases; CA19-9, carbohydrate antigen 19-9

**Fig. 4:**
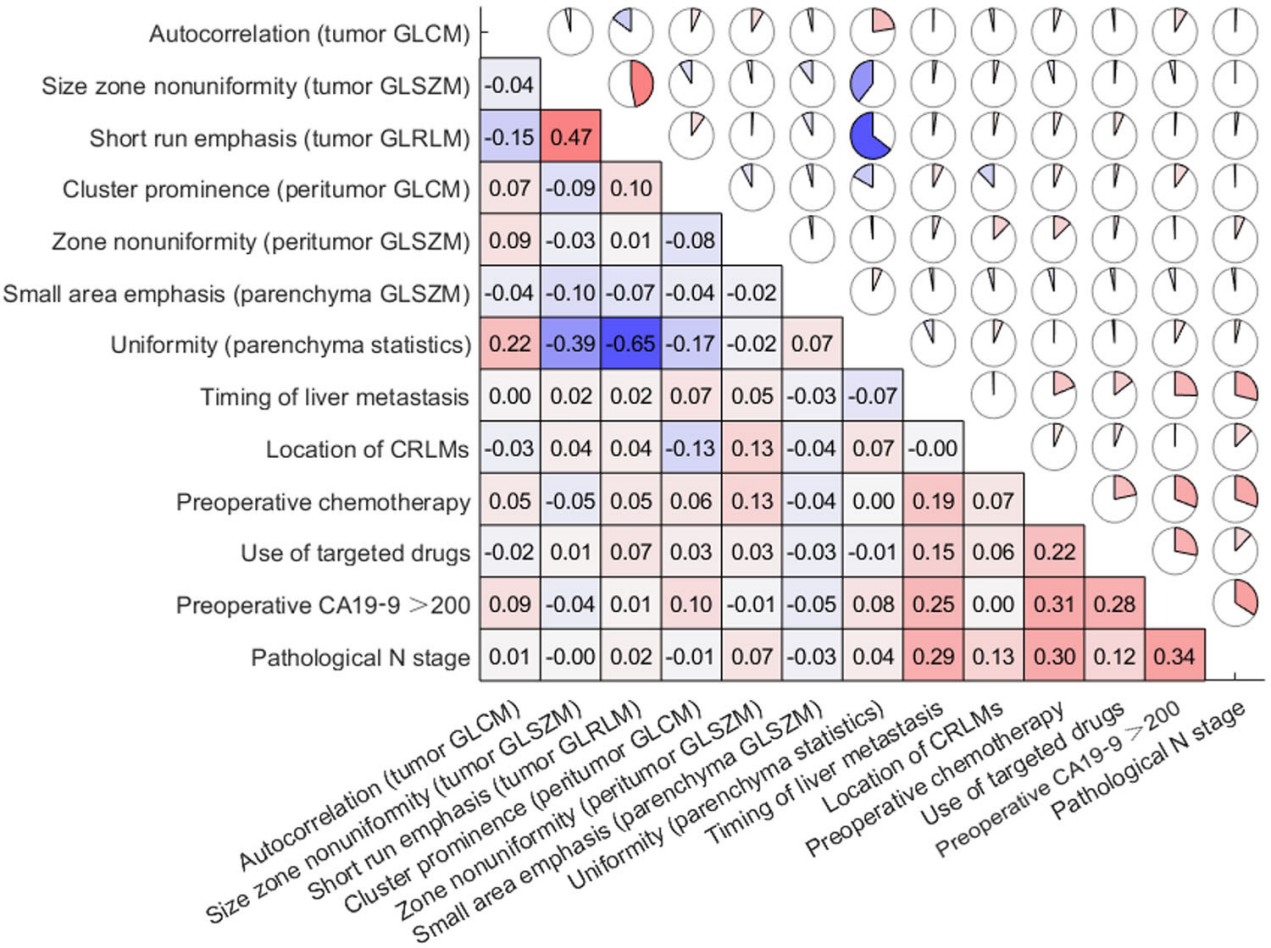
Heatmap showing the correlation degree for each pair of selected features. GLCM, gray-level co-occurrence matrix; GLSZM, gray-level size zone matrix; GLRLM, gray-level run length matrix; CRLM, colorectal cancer liver metastases; CA19-9, carbohydrate antigen 19-9

The most significant predictors were identified through LASSO, while Spearman correlation analysis was used to measure the correlation between each pair of the identified variables. Preoperative CA19-9 > 200 U/mL, pathological lymph node positivity, the location and timing of liver metastases, preoperative chemotherapy and use of targeted drugs were correlated well with intrahepatic recurrence, while the bilobar liver metastases were the strongest risk factor, showing the highest Spearman correlation coefficient (*r* = 0.29) (Fig. 3). A total of 837 radiomics features were extracted, including 162 first-order features and 675 texture features [29]. Finally, three liver tumor texture features (autocorrelation, size zone nonuniformity, short run emphasis), two peritumoral texture features (cluster prominence, zone nonuniformity), and two liver parenchyma texture features (small area emphasis, uniformity) were selected. Autocorrelation and uniformity demonstrated negative correlations with intrahepatic recurrence after liver resection, with correlation coefficients of −0.12 and −0.24, respectively. Short-run emphasis and uniformity show a strong negative correlation value of −0.65. Preoperative CA19-9 levels greater than 200 U/mL show a moderate positive correlation with pathological N staging (Fig. 4).

### Model performance and comparisons

**Fig. 5:**
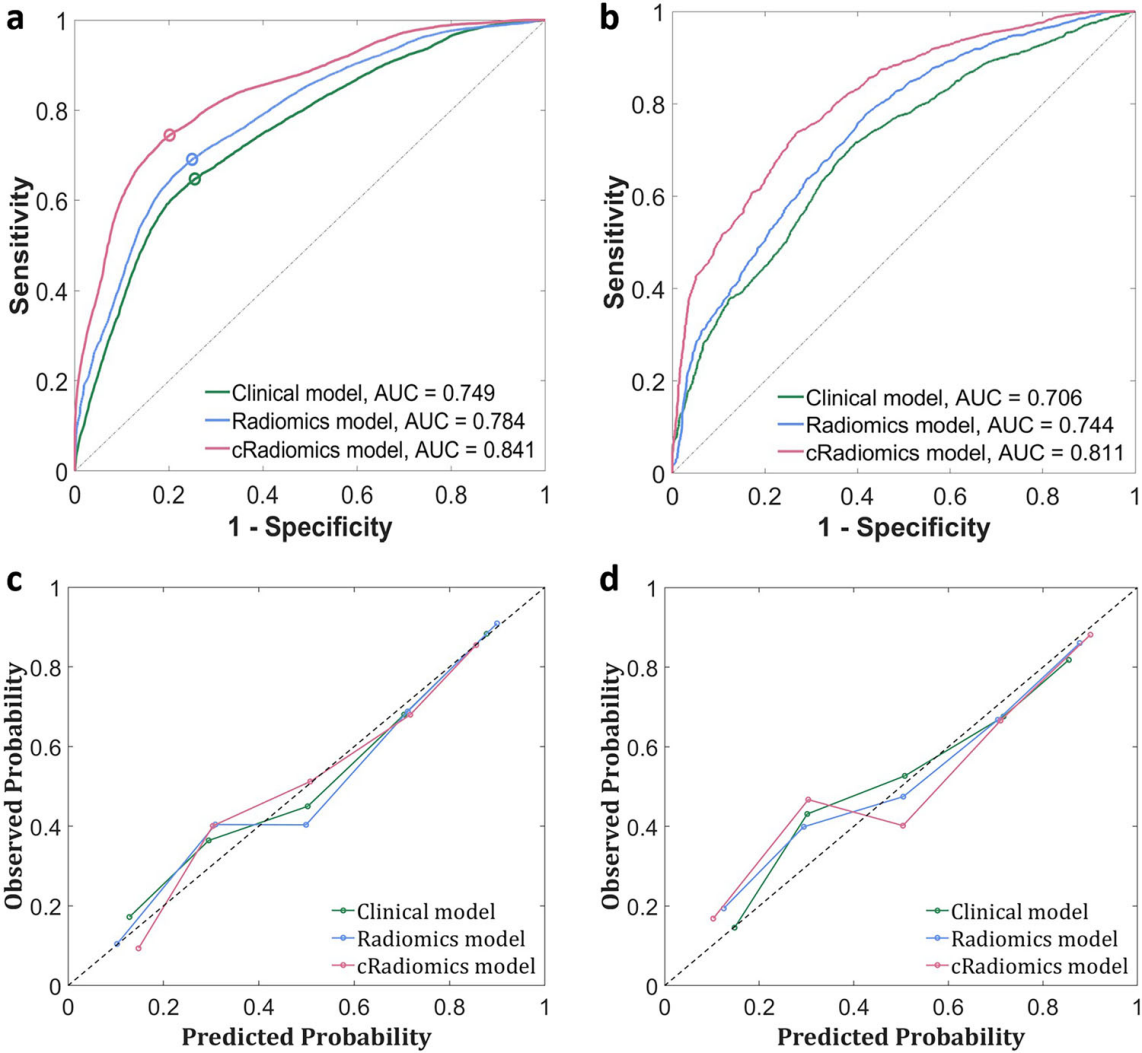
ROC and calibration curves of various models in the internal validation: **a**, **c** Present respectively the ROC and calibration curves on the training data of the main cohort (the sample size of the training data is 180 × 20, i.e., the number of patients for four training folds in each FFCV multiplied by the number of FFCV runs). **b**, **d** Correspond respectively to the ROC and calibration curves on the testing data of the main cohort (the sample size of the testing data is 44 × 20, i.e., the number of patients for one testing fold in each FFCV multiplied by the number of FFCV runs). The circle points in **a** denote the cutoff points identified according to the Youden index. ROC, receiver-operating-characteristic; AUC, area under the ROC curve; cRadiomics, clinical and radiomics feature-combined model

In internal validation, cRadiomics achieved an AUC of 0.841 (95% CI: 0.836–0.845) on the training data of the main cohort, compared to 0.784 (0.779–0.789) and 0.749 (0.744–0.754) for Radiomics and the Clinical model, respectively (*p*s < 0.01) (Fig. 5a and Table 2). Regarding calibration in this comparison, cRadiomics presented a calibration slope of 0.974, an intercept of 0.015, and a Brier score of 0.163, which were more favorable than those of the other two models, specifically 0.950, 0.023, and 0.195 for Radiomics, and 0.908, 0.055, and 0.181 for the Clinical model (Fig. 5c and Table 2). On the testing data of the main cohort, cRadiomics achieved an AUC of 0.811 (95% CI: 0.755–0.861), compared to 0.744 (0.683–0.801) and 0.706 (0.641–0.764) for Radiomics and the Clinical model, respectively (*p*s < 0.01) (Fig. 5b and Table 3). For calibration in this context, compared to the other models, cRadiomics exhibited a lower calibration slope (0.811 vs 0.832 and 0.960) and a higher intercept (0.108 vs 0.102 and 0.085), whereas its Brier score was higher (0.183 vs 0.192 and 0.204) (Fig. 5d and Table 3). Boxplots of the AUCs yielded by multiple runs of FFCV of each model demonstrated the superior performance of cRadiomics over the others (Fig. S1).

**Table 2:** Predictive performances of various models on the training data of the main cohort during internal validation

Model	ACC (95% CI)	SEN (95% CI)	SPE (95% CI)	AUC (95% CI)	Cutoff value	Calib slope	Calib intercept	Brier score
Clinical model	0.684 (0.679–0.690)	0.648 (0.640–0.655)	0.745 (0.737–0.753)	0.749 (0.744–0.754)	0.454	0.908	0.055	0.181
Radiomics model	0.714 (0.708–0.719)	0.691 (0.684–0.698)	0.751 (0.742–0.759)	0.784 (0.779–0.789) *p* < 0.001	0.443	0.950	0.023	0.195
cRadiomics model	0.765 (0.760–0.770)	0.745 (0.738–0.751)	0.799 (0.791–0.806)	0.841 (0.836–0.845) *p* < 0.001	0.441	0.974	0.015	0.163

95% CIs were computed using the Clopper–Pearson method. *p* values measure the differences of AUC values with that of the Clinical model as baseline. Cutoff values were derived from the optimal cutoff point identified based on the Youden index of the ROC curves on the training data. Calibration (Calib) slope and intercept were derived by line fitting to the sample points of the calibration curve

*ACC* accuracy, *SEN* sensitivity, *SPE* specificity, *AUC* area under the receiver operating characteristic curve, *CI* confidence interval, *cRadiomics* clinical and radiomics feature-combined model

**Table 3:** Predictive performances of various models on the testing data of the main cohort during internal validation

Model	ACC (95% CI)	SEN (95% CI)	SPE (95% CI)	AUC (95% CI)	Cutoff value	Calib slope	Calib intercept	Brier score
Clinical model	0.663 (0.599–0.727)	0.679 (0.594–0.761)	0.641 (0.537–0.738)	0.706 (0.641–0.764)	0.454	0.860	0.085	0.204
Radiomics model	0.674 (0.608–0.735)	0.674 (0.586–0.754)	0.675 (0.570–0.766)	0.744 (0.683–0.801) *p* < 0.01	0.443	0.832	0.102	0.192
cRadiomics model	0.735 (0.674–0.793)	0.738 (0.652–0.810)	0.731 (0.625–0.813)	0.811 (0.755–0.861) *p* < 0.01	0.441	0.811	0.108	0.183

95% CIs were computed using the Clopper–Pearson method. *p* values measure the differences of AUC values with that of the Clinical model as baseline. Cutoff values were derived from the optimal cutoff point identified based on the Youden index of the ROC curves on the training data of the main cohort. Calibration (Calib) slope and intercept were derived by line fitting to the sample points of the calibration curve

*ACC* accuracy, *SEN* sensitivity, *SPE* specificity, *AUC* area under the receiver operating characteristic curve, *CI* confidence interval, *cRadiomics* clinical and radiomics feature-combined model

**Fig. 6:**
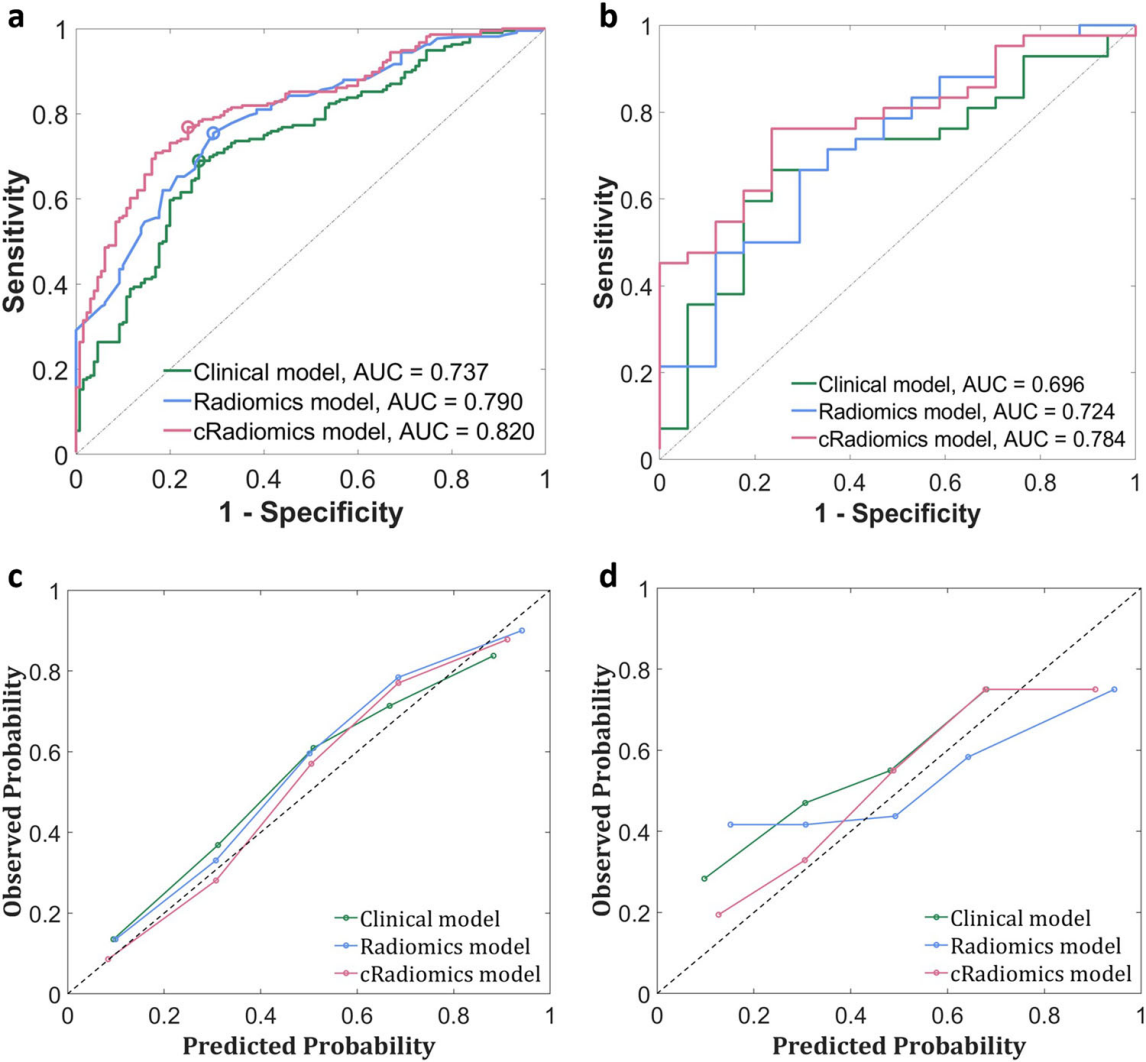
ROC and calibration curves of various models in the external validation: **a**, **c** show respectively the ROC and calibration curves on the training data of the main cohort (the sample size of the training data is 224, i.e. the total number of patients in the main cohort); **b**, **d** show respectively to the ROC and calibration curves on the testing data of the external cohort (the sample size of the testing data is 54, i.e. the total number of patients in the external cohort). The circle points in **a** denote the cutoff points identified according to the Youden index. ROC, receiver-operating-characteristic; AUC, area under the ROC curve; cRadiomics, clinical and radiomics feature-combined model

In external validation, cRadiomics achieved an AUC of 0.820 (95% CI: 0.765–0.869) on the training data of the main cohort, which was significantly higher than the other two models [0.790 (0.731–0.842) and 0.737 (0.674–0.793) for Radiomics and the Clinical model, respectively, *p*s < 0.05] (Fig. 6a and Table 4). Regarding calibration in this comparison, cRadiomics presented a calibration slope of 1.016, an intercept of 0.010, and a Brier score of 0.184, which were better than those of the other models, specifically 0.952, 0.066, and 0.210 for Radiomics, and 0.908, 0.084, and 0.224 for the Clinical model (Fig. 6c and Table 4). For testing on the external cohort, cRadiomics achieved an AUC of 0.784 (95% CI: 0.644–0.880), which was also significantly higher than those achieved by the other two models [AUCs: 0.724 (0.584–0.835) and 0.696 (0.564–0.820) for Radiomics and the Clinical model, respectively, *p*s < 0.05] (Fig. 6b and Table 5). For calibration in this context, cRadiomics presented a calibration slope of 0.784, an intercept of 0.122, and a Brier score of 0.250, which were more favorable than those of the other two [0.446, 0.294, and 0.285 for Radiomics, and 0.772, 0.211, and 0.336 for the Clinical model (Fig. 6d and Table 5).

**Table 4:** Predictive performances of various models on the training data of the main cohort during external validation

Model	ACC (95% CI)	SEN (95% CI)	SPE (95% CI)	AUC (95% CI)	Cutoff value	Calib slope	Calib intercept	Brier score
Clinical model	0.708 [159/224] (0.646–0.768)	0.690 [89/129] (0.603–0.768)	0.738 [70/95] (0.636–0.822)	0.737 (0.674–0.793)	0.464	0.908	0.084	0.224
Radiomics model	0.737 [165/224] (0.674–0.793)	0.755 [97/129] (0.668–0.824)	0.708 [67/95] (0.603–0.794)	0.790 (0.731–0.842) *p* < 0.05	0.428	0.952	0.066	0.210
cRadiomics model	0.766 [172/224] (0.707–0.821)	0.769 [99/129] (0.685–0.837)	0.762 [72/95] (0.659–0.840)	0.820 (0.765–0.869) *p* < 0.01	0.451	1.016	0.010	0.184

Data in brackets denote the raw number. 95% CIs were computed using the Clopper–Pearson method. *p* values measure the differences of AUC values with the one of the Clinical model as baseline. Cutoff values were derived from the optimal cutoff point identified by maximizing the Youden index of the ROC curves yielded by training on the main cohort for external validation. Calibration (Calib) slope and intercept were derived by line fitting to the sample points of the calibration curve

cRadiomics, clinical and radiomics feature-combined model

*ACC* accuracy, *SEN* sensitivity, *SPE* specificity, *AUC* area under the receiver-operating-characteristic curve, *CI* confidence interval

**Table 5:** Predictive performances of various models on the testing data of the external cohort during external validation

Model	ACC (95% CI)	SEN (95% CI)	SPE (95% CI)	AUC (95% CI)	Cutoff value	Calib slope	Calib intercept	Brier score
Clinical model	0.695 [38/54] (0.564–0.820)	0.667 [25/38] (0.486–0.804)	0.765 [12/16] (0.476–0.927)	0.696 (0.564–0.820)	0.464	0.772	0.211	0.336
Radiomics model	0.678 [37/54] (0.544–0.805)	0.667 [25/38] (0.486–0.804)	0.706 [11/16] (0.413–0.890)	0.724 (0.584–0.835) *p* < 0.05	0.428	0.446	0.294	0.285
cRadiomics model	0.763 [41/54] (0.624–0.865)	0.762 [29/38] (0.598–0.886)	0.765 [12/16] (0.476–0.927)	0.784 (0.644-0.880) *p* < 0.05	0.451	0.784	0.122	0.250

Data in brackets denote the raw number. 95% CIs were computed using the Clopper–Pearson method. *p* values measure the differences of AUC values with that of the Clinical model as baseline. Cutoff values were derived from the optimal cutoff point identified by maximizing the Youden index of the ROC curves yielded by training on the main cohort for external validation. Calibration (Calib) slope and intercept were derived by line fitting to the sample points of the calibration curve

*ACC* accuracy, *SEN* sensitivity, *SPE* specificity, *AUC* area under the receiver-operating-characteristic curve, *CI* confidence interval, *cRadiomics* clinical and radiomics feature-combined model

In addition to the highest AUC values and promising calibrations, cRadiomics achieved an accuracy of 0.735 (95% CI: 0.674–0.793) in the testing data of the main cohort and an accuracy of 0.763 (0.634–0.864) in the testing data of the external cohort, with a sensitivity of 0.738 (0.652–0.810) and 0.762 (0.605–0.879), and a specificity of 0.731 (0.625–0.813) and 0.765 (0.501–0.932), respectively (Tables 3 and 5). These findings suggest that the model not only accurately distinguishes between different cases but also maintains robust and reliable predictive power across different data subsets, indicating its potential for clinical application in improving clinical decision-making and enabling personalized follow-up management.

## Discussion

Despite complete resection of hepatic lesions in CRC patients, significant heterogeneity remains in clinical outcomes, particularly in recurrence and survival rates. More than half of patients develop recurrence during follow-up, with the majority occurring within the first two years [30, 31]. The Clinical Risk Score (CRS) is one of the common prognostic models for predicting long-term survival in patients with CRLM [21]. Other prognostic systems, such as Tumor Burden Score, Genetic and Morphological Evaluation score, and Comprehensive Evaluation of Relapse Risk score, incorporate a variety of clinical and molecular markers, including bilobar liver metastases, CEA and CA19-9 levels, and KRAS, NRAS, BRAF mutations [32–34]. However, as more prognostic factors are continuously identified, relying solely on these systems to assess intrahepatic recurrence risk becomes increasingly inadequate.

In this study, we developed and validated a machine learning model based on clinicopathologic parameters and radiomics features derived from liver metastases, peritumoral regions, and grossly disease-free liver parenchyma on preoperative grayscale ultrasound images. The model demonstrated promising classification and calibration performances in predicting intrahepatic recurrence within two years after curative liver resection of CRLM. In addition to the lesion, the tumor margin could reveal biological and pathological characteristics of the tumor. A 2017 international consensus guideline emphasizes that histopathologic growth patterns of CRLM—desmoplastic, pushing, and replacement—carry significant prognostic and predictive value [35]. Desmoplastic growth pattern responds well to chemotherapy, while replacement growth pattern shows a poor response to systemic treatment, suggesting a less favorable prognosis. However, definitive evaluation of the growth patterns primarily relies on postoperative histopathological assessment. Some studies achieved a high accuracy in predicting the growth patterns by applying radiomics on MRI images of CRLMs [36, 37]. Importantly, research indicated that there was a strong correlation between intrahepatic micrometastases and poorer clinical outcomes [38]. Micrometastases can lead to intrahepatic hemodynamic alterations, but are often undetectable by conventional imaging techniques. CT or MRI texture analysis is emerging as a non-invasive technique with the potential to detect subtle changes caused by micrometastases or occult CRLMs [39–41]. Similarly, in our study, two peritumoral texture features (cluster prominence, zone nonuniformity) and two liver parenchyma texture features (small area emphasis, uniformity), derived from grayscale ultrasound images, were identified to be associated with intrahepatic recurrence.

Preoperative chemotherapy and the use of targeted drugs were identified as risk factors for intrahepatic recurrence by the proposed model. It is likely due to the higher tumor burden in these patients, as univariable analysis revealed that a tumor number ≥ 3, pathological lymph node positivity, and bilobar liver metastases were significantly associated with intrahepatic recurrence. Notably, chemotherapy-related liver injuries, such as steatohepatitis and sinusoidal obstruction, can alter liver parenchyma, leading to a heterogeneous appearance on CT scans that may obscure the detection of recurrent lesions [42]. This complicates precise lesion detection in preoperative imaging and accurate localization during surgery [43].

Although we have developed a promising model to predict intrahepatic recurrence within two years after curative resection of CRLM, our study has several limitations. First, as a retrospective study, it is subject to unavoidable selection bias, which may influence the reliability of data analysis. Second, not all tumors visualized through ultrasound imaging exhibit distinct and well-defined margins, and this may lead to a reduced number of radiomics features being extracted. But ultrasound is a convenient, non-invasive, and cost-effective modality for evaluating liver lesions compared to CT or MRI. Third, the extraction of radiomics features can be influenced by various factors, including the type and setting of examination equipment, as well as the data analysis algorithms employed. Therefore, prospective, multicenter studies are necessary to validate our findings and further establish their relevance in clinical practice.

In conclusion, the cRadiomics model, integrating ultrasound radiomics and clinicopathological data, improved the prediction of intrahepatic recurrence within two years for colorectal liver metastases after curative hepatectomy, and it holds great potential to improve clinical decision-making and enable personalized management and risk-adapted follow-up for CRLM patients.

## Supplementary information

ELECTRONIC SUPPLEMENTARY MATERIAL

## Data Availability

The datasets used and/or analyzed during the current study have been deposited in the Research Data Deposit (RDD) platform (https://www.researchdata.org.cn/) under the approval number RDDA2025603829, and are available from the corresponding author upon reasonable request.

## Abbreviations

AUC: Area under the curve
CA19-9: Carbohydrate antigen 19-9
CEA: Carcinoembryonic antigen
CI: Confidence interval
cRadiomics: Clinical and radiomics feature-combined model
CRC: Colorectal cancer
CRLM: Colorectal cancer liver metastases
CT: Computed tomography
FFCV: Five-fold cross-validation
LASSO: Least absolute shrinkage and selection operator
MRI: Magnetic resonance imaging
ROC: Receiver operating characteristic
ROI: Region of interest
