# Supplementary material for: Predicting intrahepatic recurrence of colorectal cancer liver metastases after curative hepatectomy using a machine learning model with data integration of ultrasound radiomics and clinicopathological parameters
Source: Insights Imaging. 2026 Mar 16;17:72. doi: 10.1186/s13244-026-02227-2 (PMC12992848; doi:10.1186/s13244-026-02227-2)
Supplement: Supplementary file 1 — ELECTRONIC SUPPLEMENTARY MATERIAL [file 13244_2026_2227_MOESM1_ESM.pdf]

Predicting intrahepatic recurrence of colorectal cancer liver metastases after curative hepatectomy using a machine learning model with data integration of ultrasound radiomics and clinicopathological parameters

ELECTRONIC SUPPLEMENTARY MATERIAL

This supplementary material present more details on the internal and external validations of the developped machine learning models, including cross-validation schemes, performance metrics, and calibration analysis. Moreover, boxplots of the area under the receiver-operating-characteristic curve (AUC) values across twenty independent runs of the five-fold cross-validations (FFCV) of various models are included for comparison.

Model evaluation

Both internal and external validations were performed to evaluate classification performances of various models. For internal validation, twenty times of FFCV was performed on the main cohort. In each round of FFCV: The entire main cohort data were randomly stratified into five subsets, with no patient overlap among subsets and each subset containing an approximately equal number of data samples; Four out of the five subsets were used for model derivation (three for training, and the remaining one for hyperparameter selection via grid search[1]), while the remaining subset served as an internal testing set; The training-and-testing process was repeated five independent times, ensuring that each of the five subsets was used for internal

testing exactly once. After completing twenty times of FFCV, the predicted probabilities for samples involved in the training or internal testing process were aggregated together to generate a composite receiver-operating-characteristic (ROC) curve, from which a set of quantitative metrics, including accuracy, sensitivity, specificity and the area under the ROC curve (AUC), were calculated for performance evaluation and comparison. The accuracy, sensitivity, and specificity were computed using the cutoff value derived from the optimal cutoff point identified by maximizing the Youden index[2] of the ROC curves on the training data set. For external validation, all models were retrained on the entire main cohort data, with 80% of the data used for training and the remaining 20% for hyperparameter selection; the resulting models were then evaluated on the external cohort in terms of metrics same as those used in the internal validation. In addition to internal and external validations of the models' classification performances, we also evaluated the models' calibrations (the agreement between the predicted probabilities and the observed outcomes) by means of calibration plots, calibration slope/intercept, and Brier score[3]."

Model performances accross multiple runs of FFCV

Boxplots of the AUCs yielded by multiple runs of FFCV of each model are shown in Figure S1 of the supplementary material. The results demonstrated the superior performance of cRadiomics over Radiomics and the Clincal model in predicting intrahepatic recurrence of CRLM after curative hepatectomy.

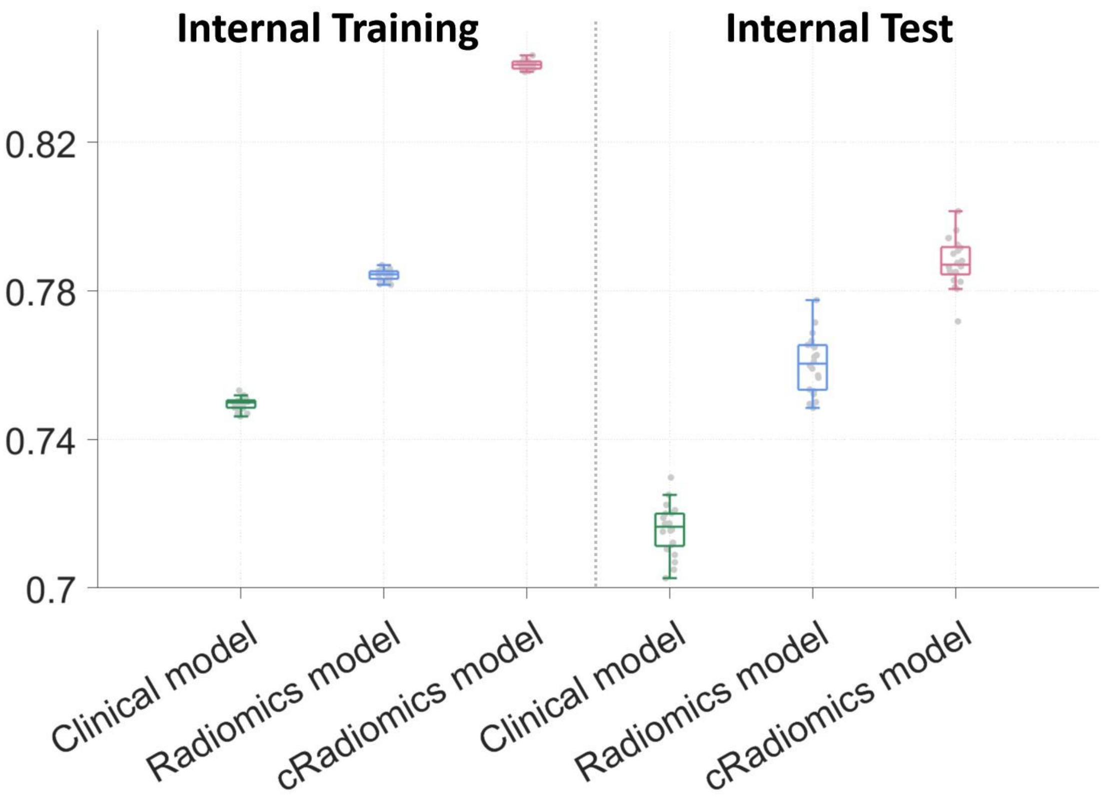

Figure S1. Boxplots of the AUC values obtained from twenty independent runs of five-fold cross-validation of various models on the main cohort data: left and right columns present respectively the training and internal testing results. The lower edge of the box denotes Q1, which is the median of the lower half of the dataset (the 25th percentile). The upper edge of the box denotes Q3, which is the median of the upper half of the dataset (the 75th percentile). The length of the box denotes the interquartile ranges within which 50% of the AUC values are located. The lines through the middle of the boxes represent the median values. The whiskers (lines extending from the box to the smallest and largest observations within 1.5 times the interquartile range from Q1 and Q3) show the range of the data outside the quartiles. cRadiomics, clinical and radiomics feature-combined model.

References:

1. Syarif I, Prugel-Bennett A, Wills G (2016) SVM Parameter Optimization using Grid Search and Genetic Algorithm to Improve Classification Performance. TELKOMNIKA (Telecommunication Computing Electronics and Control) 14(4): 1502-1509.

2. Youden WJ (1950) Index for rating diagnostic tests. Cancer 3:32-35

3. Rufibach K (2010) Use of Brier score to assess binary predictions. J Clin Epidemiol 63:938-939
